# Frequency, course and correlates of alcohol use from adolescence to young adulthood in a Swiss community survey

**DOI:** 10.1186/1471-244X-8-5

**Published:** 2008-01-17

**Authors:** Hans-Christoph Steinhausen, Susanne Eschmann, Annina Heimgartner, Christa Winkler Metzke

**Affiliations:** 1 Department of Child and Adolescent Psychiatry, University of Zurich, Neumuensterallee 9, CH 8032 Zurich, Switzerland

## Abstract

**Background:**

Few studies have analyzed the frequency of alcohol use across time from adolescence to young adulthood and its outcome in young adulthood. A Swiss longitudinal multilevel assessment project using various measures of psychopathology and psychosocial variables allowed for the study of the frequency and correlates of alcohol use so that this developmental trajectory may be better understood.

**Method:**

Alcohol use was studied by a questionnaire in a cohort of N = 593 subjects who had been assessed at three times between adolescence and young adulthood within the Zurich Psychology and Psychopathology Study (ZAPPS). Other assessment included questionnaire data measuring emotional and behavioural problems, life events, coping style, self-related cognitions, perceived parenting style and school environment, and size and efficiency of the social network.

**Results:**

The increase of alcohol use from early adolescence to young adulthood showed only a few sex-specific differences in terms of the amount of alcohol consumption and the motives to drink. In late adolescence and young adulthood, males had a higher amount of alcohol consumption and were more frequently looking for drunkenness and feeling high. Males also experienced more negative consequences of alcohol use. A subgroup of heavy or problem drinkers showed a large range of emotional and behavioural problems and further indicators of impaired psychosocial functioning both in late adolescence and young adulthood.

**Conclusion:**

This Swiss community survey documents that alcohol use is problematic in a sizeable proportion of youth and goes hand in hand with a large number of psychosocial problems.

## Background

The international trends in substance use and its determinants among youths have been analyzed in various recent reviews [1,2]. There can be no doubt that alcohol is among the leading substances used and abused by adolescents and young adults. A European prevalence study based on data collected in 26 countries found the highest rates of drunkenness, binge drinking, and alcohol consumption among British, Danish, and Irish youths. Alcohol use and misuse was much more widely reported than illicit drugs [3].

Alcohol and nicotine use contributed most strongly to the unprecedented rise in youth drug use in Britain in the mid-1990s [1]. A German prospective longitudinal study with a large representative sample of adolescents and young adults found that cumulative lifetime incidence up to age 28 of any substance abuse or dependence was 44% and that the corresponding 12-month prevalence was 24%. In this study, nicotine dependence was most frequent (25%), followed by alcohol abuse (19%) and alcohol dependence (9%). Especially younger cohorts reported significantly earlier ages at onset of abuse and dependence [4].

In Switzerland, data were collected in collaboration with the HBSC (Health Behaviour in School-Aged Children) study under the auspices of WHO [5], in the SMASH (Swiss Multicenter Adolescent Survey on Health) [6], and in the ZAPPS (Zurich Adolescent Psychology and Psychopathology Study) [7]. All three studies converge in finding increasing rates of alcohol use and cannabis, and a trend for a closing gender gap with increasing age. Smoking, drunkenness and cannabis use greatly increased in 15-year-olds in Switzerland surveyed three times between 1986 and 1998 [8].

To date, there is only a small number of longitudinal studies that have taken a developmental perspective by studying the association of adolescent substance use with adult outcome [9-12] or the escalated substance use from early to middle adolescence [13]. Within the developmental perspective of adolescent substance use, various risk factors including life stress, personality features, parental behaviour, peer influences, school characteristics, and other environmental features have been studied as can be delineated from both major reviews and empirical studies [7,13-20].

The present study is aiming at further analysis of developmental patterns of alcohol use among youths. Two major issues were studied, namely, the study of the frequencies of alcohol use including sex effects and the associations between adolescent alcohol use and other behaviours both in adolescence and in young adulthood. In the latter approach, a typology of adolescent alcohol use was employed that had been established and validated in previous work [21].

## Methods

### Subjects

Originally, the present sample is based on a cohort of 1964 pupils aged 6 to 17 who were living in the Canton of Zurich, Switzerland in 1994. The cohort was a stratified randomized sample representing the 12 counties of the canton, the school grades, and the types of school and formed the basis of the Zurich Epidemiological Study of Child and Adolescent Psychopathology (ZESCAP). A full description of details of the sampling procedure was given in a previous article [22]. The preadolescents and adolescents (aged 11 – 17 years) of the ZESCAP sample (N = 1110) provided the basic cohort of the longitudinal Zurich Adolescent Psychology and Psychopathology Study (ZAPPS).

This original cohort of 1110 subjects was studied longitudinally at three times, namely, in 1994 (time 1), 1997 (time 2) and 2001 (time 3). At each time, a multidimensional screening based on various questionnaires measuring internalizing, externalizing, total problems, depression, eating disturbance, alcohol and other drug abuse was performed. In a second stage, structured psychiatric interviews were used with those subjects who scored above the cut-off scores and with a certain number of controls scoring below the cut-off score on each screening instrument. Assessments at time 1 and predominantly also at time 2 were performed at the schools of the participants. Questionnaires had to be mailed to a smaller part of older participants at time 2 and the entire cohort at time 3 because of having left school. At each stage of the study some subjects dropped out from the sample (e.g. after leaving school and not responding to mailed questionnaires) on both the screening and the interview level.

In order to work with a full data set including all data from both the screening and the interview stages and based on a sample that still was representative for local census data in terms of age and gender composition, the final longitudinal cohort with three waves of assessment was reduced to N = 593. There was a significantly higher loss of males across time (52.5% in the original cohort, 47.9% in the longitudinal sample, 57.8% in the drop-outs, Chi^2 ^= 10.95, df = 1, p < .01). Mean age was slightly though significantly higher in the drop-outs than in the participants of the longitudinal sample (13.92 vs.13.57 years, F = 13.66, df = 1, p < .001). More importantly, multivariate analyses indicated that the amount of emotional and behavioural problems as measured by the Youth Self Report (see below) was significantly different for drop-outs as compared to participants (Wilks Lambda = .97; F = 4.89; df = 8,1082, p < .001). Drop-outs had significantly higher mean raw scores on scales measuring somatic problems (2.70 vs. 2.39, F = 4.6, df = 1, p < .05), attention problems (3.89 vs. 3.38, F = 10.7, df = 1, p < .01), delinquent behaviour (3.33 vs. 2.65, F = 27.2, p < .001), and aggressive behaviour (7.46 vs. 6.45, F = 13.5, df = 1, p < .001). Thus, there was some indication that older adolescent males with predominantly more externalizing problems were more likely to drop out from the study. However, all differences were relatively small in magnitude and became easily significant because of the large sample size.

Mean ages at the three times of the study were 13.6 (SD = 1.6), 16.6 (SD = 1.6), and 20.2 (SD = 1.7) years. The sample was composed of 284 (47.9%) males and 309 (52.1%) females. These 593 subjects were representative for the census population with regard to gender (Chi^2 ^= 2.14, df = 1, p = n.s.) and biannual age distribution of 17 – 22 years olds (Chi^2 ^= 2.67, df = 2, p = n. s.).

### Measures

The ZAPPS is based on a theoretical model in order to study conditions and processes that are essential to the mental health of growing young people as well as to the development of mental problems and disorders. A broadband questionnaire was chosen in order to obtain information on relevant behavioural and emotional problems of adolescents. Furthermore, various questionnaires dealing with depression, abnormal eating behaviour, and substance abuse were also included. In order to analyze potential risk, compensatory, vulnerability, and protective factors of psychopathology [23], life events were hypothetically seen as stressors, and various psychosocial variables including coping, self-related cognitions, and features of the social network were regarded as moderating factors with regard to behavioural and emotional problems.

Questionnaires were filled out confidentially by the subjects during school hours in 1994 and had to be mailed in 1997 and 2001. All questionnaires reflect raw scores and are positively keyed, i.e. high scores represent high expression of the content of the scale. All scales showed good to excellent reliability. A list of Alpha coefficients may be obtained from the authors.

#### Substance Use Questionaire (SUQ)

The questionnaire was designed by Müller and Abnet [24] in collaboration with the World Health Organization for a nationwide Swiss survey. It covers 22 items that deal both with the consumption of legal drugs and illegal drugs. Eight items deal with alcohol use by the respondent. The response format varied for the different items. Alcohol use was assessed via a general introductory question whether or not the adolescent had ever consumed alcohol (0 = no consumption, 1 = only a sip, 2 = an entire glass or more) and a detailed list of various alcoholic beverages with a response format ranging from 0 (no consumption) to 5 (daily consumption). Various subgroups were identified and a typology of adolescent alcohol use was validated [21]. The four types comprise abstainers, social drinkers, heavy drinkers, and problem drinkers. These four types are also relevant for the present study. Abstainers were negative on all items. Social drinkers were defined by three positive responses to the following items: I drink when I am in the company of friends/on the occasion of a family celebration/at a party. Heavy drinkers were defined by two positive responses to the following items: I drink until I feel high/until I get drunk. Problem drinkers had to respond positively to the following two items: I drink when I feel lonely/when I feel bad and having a problem.

#### Young Adult Self Report (YASR)

With the exception of the subscale measuring social problems and the inclusion of the subscale measuring intrusiveness the YASR [25] consists of the following primary subscales: socially withdrawn, somatic complaints, anxious/depressed, intrusiveness, thought problems, attention problems, delinquent behaviour, and aggressive behaviour. Two second-order scales reflecting internalizing and externalizing can be calculated.

#### Life Event Scale (LES)

A total of 36 items were chosen from pre-existing questionnaires on life events. The time frame was defined as the twelve months prior to filling out the questionnaire. Beside frequencies of life events, a total impact score was calculated. This was based on a scale attached to each item ranging from -2 to +2 and indicating how unpleasant or pleasant the respective event was [26].

#### Coping Capacities (CC)

Our modified version of the German Coping Across Situations Questionnaire [27] addresses coping in four problem areas with school, parents, peers, and the opposite sex. Factor analysis resulted in two scales measuring active coping and avoidant behaviour.

#### Self-Related Cognitions (SRC)

The ten-item scale for the measurement of self-esteem by Rosenberg [28] and items from a German questionnaire assessing self-awareness [29] were further included into the questionnaire. The latter scale assesses introspective capacities for one's feelings, actions, and past.

### Statistical analyses

All questionnaire scores represent raw scores. Data were analysed by use of the 14^th ^version of the SPSS (2006) program. Sex differences in alcohol use were analyzed by Chi-square tests. Comparisons between the various types of alcohol use were based on univariate and multivariate analyses of covariance (ANCOVA and MANCOVA) with sex and age as the controlled covariates. Stability of types of alcohol use across time was tested by the McNemar test.

## Results

**Figure 1:**
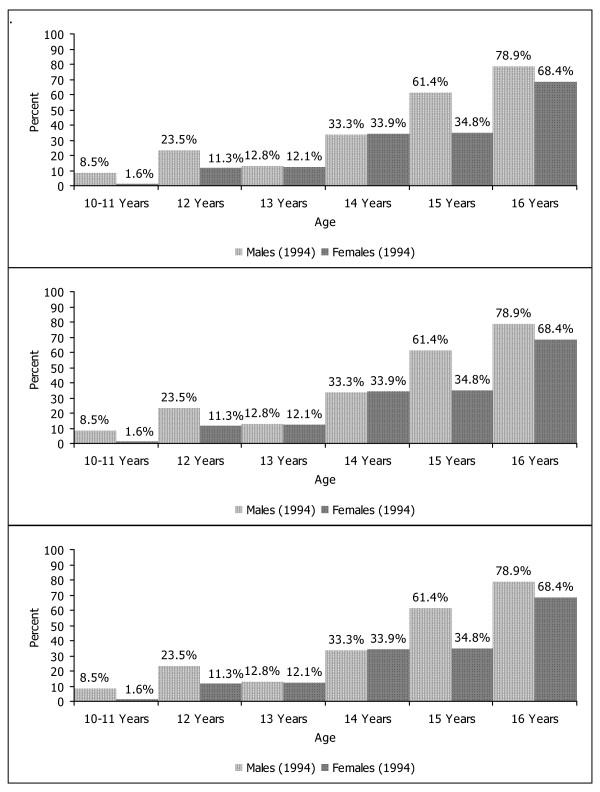
Frequencies of alcohol consumption (≥1 glass of alcohol ever) at three times.

**Figure 2:**
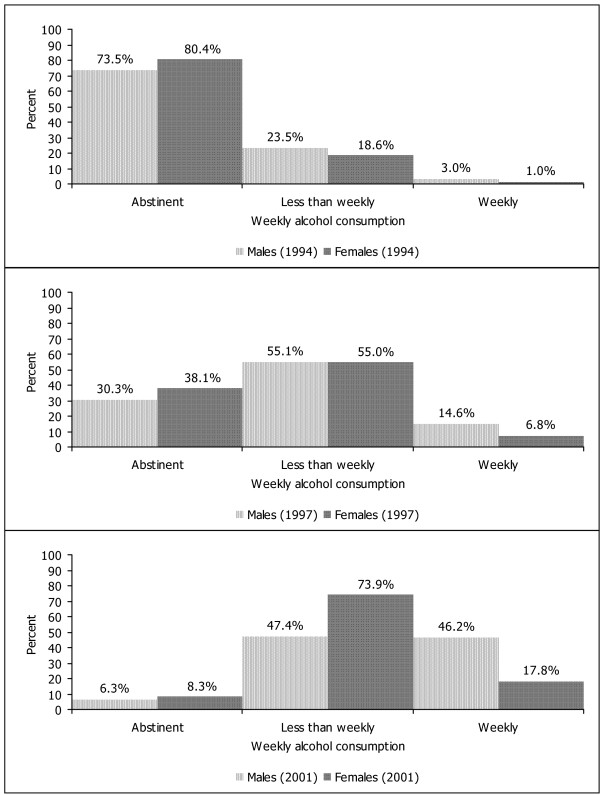
Weekly alcohol consumption at three times.

Figure 1 displays the development of any alcohol consumption across time. Around the age of 15 years more than half of the sample had drunken at least a glass of alcohol ever. Only at time 1 there were significant sex differences with males more likely to have consumed alcohol (Chi^2 ^= 6.33, df = 2, p < .05). Weekly alcohol consumption is shown in Figure 2. Whereas around two per cent of the subjects showed weekly alcohol consumption in early adolescence at time 1, almost a third of young adults engaged in weekly alcohol consumption at time 3. There was only a trend for an excess of males at time 1 (Chi^2 ^= 5.40, df = 2, p = .07) which became a significant difference at time 2 (Chi^2 ^= 9.90, df = 2, p < .01) and time 3 (Chi^2 ^= 49.98, df = 2, p < .001).

**Figure 3:**
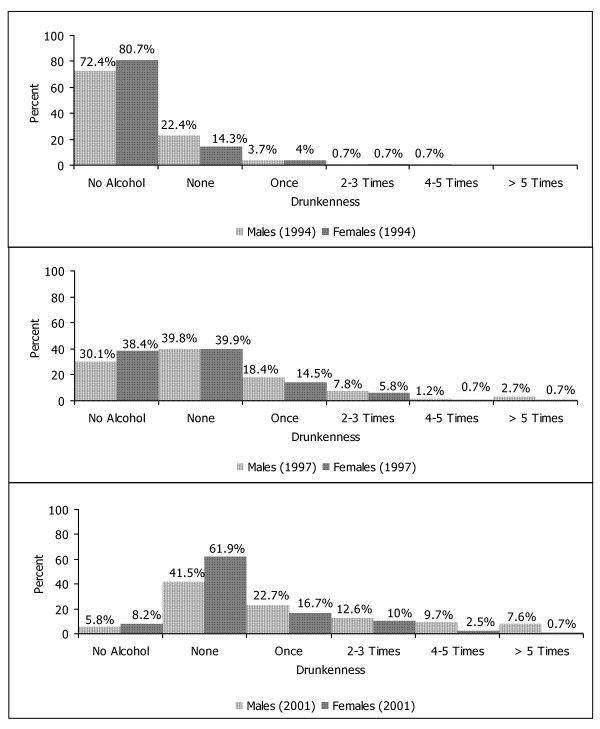
Frequencies of drunkenness during the last two months at three times.

**Figure 4:**
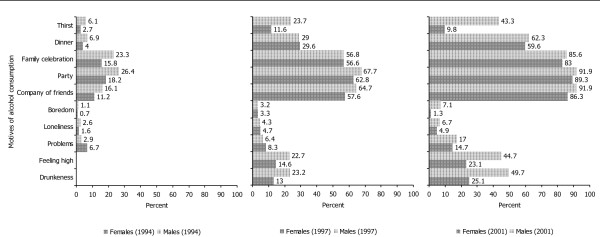
Motives of alcohol consumption at three times ("Feeling high" & "Drunkeness" were not asked at time 1).

Data on the frequencies of drunkenness during the last two months are shown in Figure 3. There were no sex significant differences at times 1 and 2 in contrast to time 3 with more males having been drunk (52.6 vs. 29.9 per cent, Chi^2 ^= 42.59, df = 4, p < .001). Furthermore, motives of drinking showed only few different distributions across time and for the two sexes as shown in Figure 4. At all three times, social events predominated among the various motives. However, there was an increasing proportion of young people who consumed alcohol in order to get drunk or feel high or when encountering problems. Both sexes did not show significant differences in alcohol consumption when encountering problems at all three times. On the other hand, more males than females drank in order to get drunk at time 2 (Chi^2 ^= 7.73, df = 2, p = .05) and at time 3 (Chi^2 ^= 40.38, df = 2, p < .001) or to feel high at time 3 (Chi^2 ^= 31.14, df = 2, p < .001).

**Figure 5:**
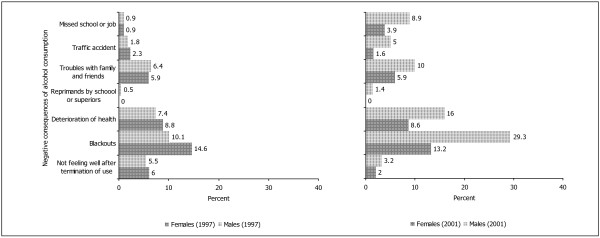
Negative consequences of alcohol consumption at times 2 and 3.

The negative consequences of alcohol use are shown in Figure 5 for times 2 and 3 only because they were not yet apparent at time 1. At both times, blackouts were experienced most frequently followed by deterioration of health, and troubles with family and friends. There were no significant sex differences at time 2. In young adulthood at time 3, the frequencies of negative consequences were increasing particularly for males. At time 3, more males than females reported to have been encountering a blackout (Chi^2 ^= 23.77, df = 2, p < .001), some deterioration of health status (Chi^2 ^= 7.62, df = 1, p < .01), to have received reprimands from school or superiors (Chi^2 ^= 4.36, df = 1, p < .05), to have been close to or definitely been involved in an accident (Chi^2 ^= 7.51, df = 2, p < .05), and to have missed school or job due to alcohol consumption (Chi^2 ^= 6.09, df = 1, p < .05).

The four different types of adolescent alcohol use were identified only at times 2 and 3 because the subjects were still too young at time 1. There was a rather small number of either heavy or problem drinkers so that the two groups were combined. At time 2 in late adolescence there were 198 (33.4%) abstainers, 363 (61.2%) social drinkers, and 32 (5.4%) heavy or problem drinkers who, at the same time, were also engaged in social drinking. At time 3 in young adulthood, there were only 46 (7.6%) abstainers, but 505 (85.2%) social drinkers and 42 (7.1%) heavy or problem drinkers. Thus, both the numbers of social drinkers and heavy or problem drinkers increased over time. The changes across time were statistically highly significant for the three groups (Mc Nemar-Bowker Chi^2 ^= 134.49, df = 3, p < .001).

The three types of adolescent alcohol use were compared at times 2 and 3 with regard to emotional and behavioural problems and further psychosocial correlates. Findings for emotional and behavioural problems at time 2 are shown in Table 1. Differences for the eight primary scales of the YSR were highly statistically different (Wilks Lambda = 0.76; F = 10.47; df = 16,1162; p < .001). There was an almost uniform pattern with the heavy or problem drinkers showing more problems than the two other groups of either abstainers or social drinkers. The former group had more somatic complaints, was more anxious depressed, had more thought problems and more attention problems, was more aggressive and showed more delinquent behaviour, and as a consequence also more internalizing and externalizing problems and a higher total score than the two other groups. Among the two latter groups there was less of a differentiation, with the social drinkers having an intermediate position between heavy or problem drinkers and abstainers with regard to aggressive and delinquent behaviours and as a consequence also externalizing problems.

**Table 1:** Comparison of Emotional and Behavioral Problems of the Three Subsamples at Time 2 in 1997

	Abstainers (0)		Social drinkers (1)		Heavy and Problem drinkers (2)		F	
	Total (N = 198)		Total (N = 363)		Total (N = 32)		Groups	Post-hoc
	Mean	SD	Mean	SD	Mean	SD	(df = 2)	Scheffé Test
Social Withdrawn	2.96	2.39	2.60	2.22	3.93	2.77	7.25**	2 > 1
Somatic Complaints	2.28	2.14	2.63	2.35	4.21	2.82	12.20***	2 > 0, 1
Anxious/Depressed	5.19	4.21	5.11	4.35	8.31	5.45	8.10***	2 > 0, 1
Social Problems	2.01	2.10	1.38	1.69	2.14	2.70	8.29***	0 > 1
Thought Problems	1.86	1.83	1.97	1.92	3.81	2.51	15.31***	2 > 0, 1
Attention Problems	3.57	2.57	3.65	2.49	6.24	4.12	15.31***	2 > 0, 1
Aggressive Behavior	5.77	3.90	6.91	4.14	10.18	4.52	22.58***	2 > 1 > 0
Delinquent Behavior	2.41	1.85	3.68	2.25	6.42	2.46	61.42***	2 > 1 > 0
								
Internalizing	10.07	7.10	9.92	7.32	15.72	8.93	9.51***	2 > 0, 1
Externalizing	8.18	5.21	10.59	5.64	16.60	5.85	43.21***	2 > 1 > 0
								
Total Score	29.91	17.01	32.36	17.26	51.75	21.72	23.10***	2 > 0, 1

*p < .05 **p < .01 ***p < .001

Findings from comparisons of further psychosocial correlates are presented in Table 2. Again the heavy and problem drinkers stand out by a large number of statistically significant differences (Wilks Lambda = 0.81; F = 3.42; df = 38,1140; p < .001). Compared to the two other groups they had experienced a higher number and a more negative impact of life events, used less active and more avoidant coping, had less self-esteem but more self-awareness, felt less accepted and more rejected by both parents, and experienced more controlling teachers and less possibilities to participate at school. Again, the social drinkers had an intermediate position between the two other groups with regard to the number and impact of life events, paternal acceptance, and possibilities to participate at school. The abstainers felt less parental control, less performance stress, and less peer acceptance than the social drinkers.

**Table 2:** Comparison of Psychosocial Correlates of the Three Subsamples at Time 2 in 1997

	Abstainers (0)		Social drinkers (1)		Heavy and Problem drinkers (2)		F	
	Total (N = 198)		Total (N = 363)		Total (N = 32)		Groups	Post-hoc
	Mean	SD	Mean	SD	Mean	SD	(df = 2)	Scheffé Test
Number of Live Events	4.39	2.94	5.82	3.34	8.08	3.80	20.90***	2 > 1 > 0
Life Events Impact	-5.12	4.21	-6.32	4.84	-8.81	5.27	10.12***	2 < 1 < 0
Active Coping	4.92	1.16	4.91	1.18	4.16	1.43	7.06**	2 < 1, 0
Avoidant Coping	2.57	1.43	2.71	1.41	3.36	1.13	6.90**	2 > 1, 0
Self-Esteem	27.58	5.85	27.74	5.96	22.42	6.72	12.28***	2 < 1, 0
Self-Awareness	19.89	5.37	20.05	5.55	22.81	5.31	4.08*	2 > 1, 0
Maternal Acceptance	28.48	5.50	27.30	5.95	24.44	6.59	7.77***	2 < 1, 0
Maternal Rejection	5.32	4.12	5.69	4.45	7.71	4.39	7.15**	2 > 1, 0
Maternal Control	10.56	3.90	9.58	3.84	9.19	3.75	0.57	1 < 0
Paternal Acceptance	26.53	5.69	24.72	6.90	21.44	7.52	9.46***	2 < 1 < 0
Paternal Rejection	5.25	3.57	5.75	4.28	8.14	5.26	9.37***	2 > 1, 0
Paternal Control	9.61	3.77	8.58	3.65	8.20	5.03	0.33	1 < 0
Competition at School	7.56	4.58	8.06	4.83	9.69	5.04	3.51*	-
Controlling Teachers	13.27	5.77	13.69	5.91	17.22	6.97	11.47***	2 > 1, 0
Possibilities to Participate	16.57	3.65	15.36	4.29	12.44	5.28	12.73***	2 < 1 < 0
Performance Stress	7.61	3.58	8.55	3.87	9.06	4.01	2.91	1 > 0
Peer Acceptance	14.27	3.50	15.24	3.15	14.56	3.93	4.35*	1 > 0
Size of Social Network	21.84	5.95	21.05	6.30	19.41	6.14	1.25	-
Efficiency of Social Network	21.84	3.23	21.62	3.16	20.46	3.60	2.22	-

*p < .05 **p < .01 ***p < .001

Further comparisons of emotional and behavioural problems among the three groups at time 3 are shown in Table 3. The findings from time 2 in late adolescence are clearly replicated in young adulthood. Again, the heavy or problem drinkers showed significantly more emotional and behavioural problems than the other two groups across all primary scales measuring social withdrawn, anxious/depressed, intrusiveness, attention problems, aggressive behaviour, and delinquent behaviour. As a consequence, they had significantly more internalizing problems and a higher total score than the social drinkers and more externalizing problems than the two other groups. The social drinkers were less social withdrawn, less anxious/depressed, and less inattentive than the abstainers and the latter had the lowest score with regard to attention problems and delinquent behaviours.

**Table 3:** Comparison of Emotional and Behavioral Problems of the Three Subsamples at Time 3 in 2001

	Abstainers (0)		Social drinkers (1)		Heavy and Problem drinkers (2)		F	
	Total (N = 46)		Total (N = 505)		Total (N = 42)		Groups	Post-hoc
	Mean	SD	Mean	SD	Mean	SD	(df = 2)	Scheffé Test
Social Withdrawn	4.48	3.25	1.97	1.87	2.35	2.18	35.29***	2, 1 < 0
Somatic Complaints	2.58	2.56	2.25	2.36	3.22	3.11	5.88**	2 > 1
Anxious/Depressed	8.20	5.94	5.95	4.86	8.35	6.52	9.14***	2, 0 > 1
Intrusiveness	1.96	1.94	2.28	1.94	3.33	2.00	4.73**	2 > 0, 1
Thought Problems	0.40	0.88	0.23	0.65	0.79	1.38	10.93***	2 > 1
Attention Problems	2.76	2.38	1.88	1.90	3.15	2.25	11.06***	2 > 1 > 0
Aggressive Behavior	2.71	2.56	2.48	2.30	3.97	3.26	6.98**	2 > 1, 0
Delinquent Behavior	0.87	1.32	1.77	1.98	4.57	3.14	36.15***	2 > 1 > 0
								
Internalizing	12.68	8.49	7.92	6.15	10.70	7.91	14.68***	2, 0 > 1
Externalizing	5.54	4.54	6.54	4.75	11.88	6.42	20.93***	2 > 0, 1
								
Total Score	36.74	22.81	28.66	17.20	44.08	23.76	16.78***	2, 0 > 1

*p < .05 **p < .01 ***p < .001

Because at time 3questionnaires were mailed, instead of administered at school, the number of psychosocial variables that were assessed in young adulthood was more limited than at time 1 and 2. Findings are shown in Table 4. The multivariate analysis resulted in highly statistically significant differences (Wilks Lambda = 0.91; F = 3.41; df = 16,1162; p < .001). Across the set of variables the group of heavy or problem drinkers again stood out by having experienced a higher number of life events, and a less efficient social network than the two contrast groups. In terms of the greater negative impact of life events, the higher score of avoidant coping, and the smaller size of the social network they differed only from the social drinkers but not from the abstainers and together with the abstainers they were scoring lower than the social drinkers on scales measuring active coping and self-esteem.

**Table 4:** Comparison of Psychosocial Correlates of the Three Subsamples at Time 3 in 2001

	Abstainers (0)		Social drinkers (1)		Heavy and Problem drinkers (2)		F	
	Total (N = 46)		Total (N = 505)		Total (N = 42)		Groups	Post-hoc
	Mean	SD	Mean	SD	Mean	SD	(df = 2)	Scheffé Test
Number of Live Events	4.52	3.43	5.04	3.29	6.48	4.36	4.34*	2 > 0, 1
Life Events Impact	-5.32	4.60	-5.86	4.81	-7.83	7.06	4.54*	2 > 1
Active Coping	5.06	1.16	5.45	0.97	5.02	1.03	5.18**	2, 0 < 1
Avoidant Coping	2.87	1.18	2.62	1.07	3.11	1.16	4.73**	2 > 1
Self-Esteem	24.00	6.27	26.60	5.04	24.06	6.02	10.26***	2, 0 < 1
Self-Awareness	19.48	5.40	18.81	4.90	18.94	5.45	0.83	-
Size of Social Network	21.69	6.88	22.43	7.07	18.60	5.33	4.27*	2 < 1
Efficiency of Social Network	21.94	3.85	22.83	3.23	20.01	3.56	11.30***	2 < 0, 1

*p < .05 **p < .01 ***p < .001

## Discussion

The findings of the present study are based on longitudinal assessments in a representative cohort of young Swiss people who were assessed three times for alcohol consumption and further psychosocial parameters between early adolescence and young adulthood. In a first step, the development of alcohol use including frequencies, motives, and consequences were studied. From a starting point of a small minority of 8.5% of males and 1.6% of females who had consumed at least a single glass of alcohol at the age of 10 to 11 years, the proportion of youngsters consuming alcohol continuously progressed across time with three quarters having experienced alcohol use around the age of 16 years. Similarly, weekly consumption of alcohol progressed continuously over time with a third of adults drinking weekly and a clear male dominance with regard to the amount of alcohol consumption.

Whereas these findings represent developmental patterns with, so far, no clear indication of abnormality, there were some more worrisome results when focusing on the frequencies of drunkenness, the motives and the consequences of drinking. Drunkenness was still a rare event in early adolescence accounting only for some 5 percent of the participants of the survey, whereas a third of males and 21 percent of females in late adolescence and more than half of the males and slightly less than half of the females in young adulthood had been drunk at least once. In general, social events clearly predominated among the motives in the cohort. However, more than 20 percent of males and 13 to 15 percent of females drank in late adolescence in order to get drunk or feel high, and these figures increased to 45 to 50 percent in young adult males and to around a quarter in young adult females. The apparent male preponderance in these motives was not observed with regard to drinking when encountering problems at any time. The figures of this motive doubled from late adolescence to young adulthood up to a maximum of 17 percent of young adult males. Among various negative consequences, blackouts and deteriorations of health were noticed up to a maximum of 15 percent in adolescent females and 29 percent of young adult males. In summary, these findings match conclusions from other recent Swiss surveys about a worrisome pattern of alcohol use among young people in the country, i.e. the HBSC (Health Behaviour in School-Aged Children) study under the auspices of WHO [5], the SMASH (Swiss Multicenter Adolescent Survey on Health) [6], and the repeated surveys in 15 year olds [8].

From these descriptive findings in the entire cohort, it had to be delineated that there was a sizeable subgroup of youths with highly problematic alcohol use. Based on previous work of the authors on the identification and validation of different types of adolescent alcohol use [21] it was, thus, decided to study these types in more detail by analyzing the associations of type of alcohol use with emotional and behavioural problems and further psychosocial parameters which form the theoretical frame of the ZAPPS. Due to rather low frequencies the two types of heavy and problem drinkers had to be collapsed into a single combined group. First, it was found that this group had an increase from 5.4% in late adolescence to 7.1% in young adulthood indicating that persisting careers of problematic alcohol use start early in life. Secondly, at both times of the assessment in late adolescence and young adulthood, this group of heavy and problem drinkers was clearly different from the two contrast groups of abstainers and social drinkers.

In this risk group compared to the two other groups there were stronger associations with emotional and behavioural problems and other psychosocial abnormalities including the experience of more life events and, particularly, more negative impact of these events, and abnormalities of personality including the use of more inadequate coping styles, less self-esteem, and greater self-awareness. In addition, this problematic risk group perceived less positive parenting style and a less attractive school environment during late adolescence, and a smaller and less efficient social network in young adulthood. The two other groups of abstainers and social drinkers were indistinguishable in many domains, and only in some domains the social drinkers had an intermediate position between the risk group and the abstainers. Interestingly, in a few domains the abstainers were more similar to the heavy and problem drinkers than to the social drinkers.

## Conclusion

The heavy and problem drinkers stood out as a clearly most abnormal group in terms of its psychosocial characteristics. One may well conclude that their alcohol use was both a way of reflecting and contributing to their life that was marked by a higher amount of psychosocial stressors and deficits in coping and support. These findings converge with other reports obtained with other samples in different regions and some major recent reviews. Within a developmental perspective, all these publications have stressed the importance of various risk factors including life stress, inefficient coping, non-supportive parental behaviour, inadequate school characteristics, and other environmental features as contributing factors to adolescent substance abuse [7,13-19].

In terms of limitations, it should be noted that the longitudinal sample was not completely representative of the original cohort because slightly older males with more externalizing problems were more likely to drop out from the longitudinal study. Thus, one may argue that particularly the findings on gender and associated behavioural problems represent a rather conservative picture. However, this limitation is counterbalanced by the fact that the longitudinal sample was representative in terms of age and gender composition according to local census data. Furthermore, the present study was based on a survey that looked for indicators of problematic alcohol use but was not in a position to come up with clinically based diagnoses of alcohol abuse. Thus, comparisons with other European studies reporting rates of alcohol abuse with different assessment methods [4] are impossible. On the other hand, the present study provides further evidence that problematic alcohol use among youths is frequent and associated with a variety of mental and psychosocial problems. Early prevention in adolescence is clearly warranted.

## Competing interests

The author(s) declare that they have no competing interests.

## Authors' contributions

HCS designed the study and drafted the manuscript. SE and AH performed the statistical analyses. CWM participated in the design and coordination of the study. All authors read and approved the final manuscript.

## Pre-publication history

The pre-publication history for this paper can be accessed here:



## References

[B1] Gilvarry E (2000). Substance abuse in young people. J Child Psychol Psychiatry.

[B2] Glantz MD, Leshner AI (2000). Drug abuse and developmental psychopathology. Dev Psychopathol.

[B3] Hibell B, Andersson B, Bjarnason T, Kokkevi A, Morgan M, Narusk A, Sweden: The Swedish Council for Information on Alcohol and other drugs. (CAN) / The Pompidou Group at the Council of Europe. (1997). The 1995 ESPAD report: Alcohol and other drug use among students in 26 European countries..

[B4] Perkonigg A, Pfister H, Höfler M, Fröhlich C, Zimmermann P, Lieb R, Wittchen HU (2006). Substance Use and Substance Use Disorders in a Community Sample of Adolescents and Young Adults: Incidence, Age Effecs and Patterns of Use. European Addiction Research.

[B5] Schmid H, Delgrande J, Kuntsche EN, Kuendig H (2003). Trends in the consumption of psycho-active substances among pupils in Switzerland - Selected findings from a study under the auspices of WHO.. Research report No 39 (in German).

[B6] Narring F, Tschumper A, Inderwildi Bonivento L, Jeannin A, Addor V, Bütikofer A (2004). SMASH 2002: Swiss multicenter adolescent study on health 2002..

[B7] Steinhausen HC, Winkler Metzke C (1998). Frequency and correlates of substance use among preadolescents and adolescents in a Swiss epidemiological study. J Child Psychol Psychiatry.

[B8] Kuntsche EN (2004). Progression of a general substance use pattern among adolescents in Switzerland? Investigating the relationship between alcohol, tobacco, and cannabis use over a 12-year period. Eur Addict Res.

[B9] McGue M, Iacono WG (2005). The association of early adolescent problem behavior with adult psychopathology. Am J Psychiatry.

[B10] Flory K, Lynam D, Milich R, Leukefeld C, Clayton R (2004). Early adolescent through young adult alcohol and marijuana use trajectories: early predictors, young adult outcomes, and predictive utility. Dev Psychopathol.

[B11] Rohde P, Lewinsohn PM, Kahler CW, Seeley JR, Brown RA (2001). Natural course of alcohol use disorders from adolescence to young adulthood. J Am Acad Child Adolesc Psychiatry.

[B12] Patton GC, Coffey C, Lynskey MT, Reid S, Hemphill S, Carlin JB, Hall W (2007). Trajectories of adolescent alcohol and cannabis use into young adulthood. Addiction.

[B13] Wills TA, McNamara G, Vaccaro D, Hirky AE (1996). Escalated substance use: a longitudinal grouping analysis from early to middle adolescence. Journal of Abnormal Psychology.

[B14] Lifrak PD, McKay JR, Rostain A, Alterman AI, O'Brien CP (1997). Relationship of perceived competencies, perceived social support, and gender to substance use in young adolescents. J Am Acad Child Adolesc Psychiatry.

[B15] Webb A, Morey J, Thompsen W, Butler C, Barber M, Fraser WI (2003). Prevalence of austistic spectrum disorder in children attending mainstream schools in a welsh education authority. Developmental Medicine and Child Neurology.

[B16] Weinberg NZ, Glantz MD (1999). Child psychopathology risk factors for drug abuse: overview. J Clin Child Psychol.

[B17] Petraitis J, Flay BR, Miller TQ, Torpy EJ, Greiner B (1998). Illicit substance use among adolescents: a matrix of prospective predictors. Subst Use Misuse.

[B18] Hawkins JD, Catalano RF, Miller JY (1992). Risk and protective factors for alcohol and other drug problems in adolescence and early adulthood: implications for substance abuse prevention. Psychol Bull.

[B19] Pires P, Jenkins JM (2007). A growth curve analysis of the joint influences of parenting affect, child characteristics and deviant peers on adolescent illicit drug use. Journal of Youth and Adolescence.

[B20] Wells JE, Horwood LJ, Fergusson DM (2004). Drinking patterns in mid-adolescence and psychosocial outcomes in late adolescence and early adulthood. Addiction.

[B21] Steinhausen HC, Metzke CW (2003). The validity of adolescent types of alcohol use. J Child Psychol Psychiatry.

[B22] Steinhausen HC, Winkler Metzke C, Meier M, Kannenberg R (1998). Prevalence of child and adolescent psychiatric disorders: the Zurich Epidemiological Study. Acta Psychiatr Scand.

[B23] Steinhausen HC, Winkler Metzke C (2001). Risk, Compensatory, Vulnerability, and Protective Factors Influencing Mental Health in Adolescence. Journal of Youth and Adolescence.

[B24] Müller R, Abbet JP (1991). Veränderung im Konsum legaler und illegaler Drogen bei Jugendlichen. Ergebnisse einer Trenduntersuchung bei 11-16 jährigen Schülern unter Schirmherrschaft der Weltgesundheitsorganisation (WHO Europe). [Changing trends in the consumption of legal and illegal drugs by 11-16-year-old adolescent pupils. Findings from a study conducted under the auspices of the World Health Organisation (WHO Europe).

[B25] Achenbach TM (1997). Manual for the Young Adult Self Report and  and Young Adult Behavior Checklist..

[B26] Steinhausen HC, Winkler Metzke C (2001). Die Zürcher Lebensereignis-Liste (ZLEL): Ergebnisse einer epidemiologischen Untersuchung. [The Zurich life event list (ZLEL): Findings from an epidemiological study]. Kindheit und Entwicklung.

[B27] Seiffge-Krenke I (1989). Bewältigung alltäglicher Problemsituationen: Ein Coping-Fragebogen für Jugendliche [Coping with everyday problem situations: A coping questionnaire for adolescents]. Zeitschrift für Differentielle und Diagnostische Psychologie.

[B28] Rosenberg M (1965). Society and the adolescent self-image. Society and the Adolescent Self-Image.

[B29] Filipp SH, Freudenberg E (1989). Der Fragebogen zur Erfassung dispositionaler Selbstaufmerksamkeit [Questionnaire for the assessment of dispositional self—awareness]. Der Fragebogen zur Erfassung dispositionaler Selbstaufmerksamkeit [Questionnaire for the Assessment of Dispositional Self—Awareness] (in German).

