# Correction: Frequency, course and correlates of alcohol use from adolescence to young adulthood in a Swiss community survey

**DOI:** 10.1186/1471-244X-10-15

**Published:** 2010-02-18

**Authors:** Hans-Christoph Steinhausen, Susanne Eschmann, Annina Heimgartner, Christa Winkler Metzke

**Affiliations:** 1 Department of Child and Adolescent Psychiatry, University of Zurich, Neumuensterallee 9, CH 8032 Zurich, Switzerland

## Correction

After the publication in this journal [1] we became aware of the fact that the article contained wrong data in figure 1. This correction contains the revised figure 1. We regret any inconvenience that this inaccuracy in the presentation of the figure might have caused.

**Figure 1:**
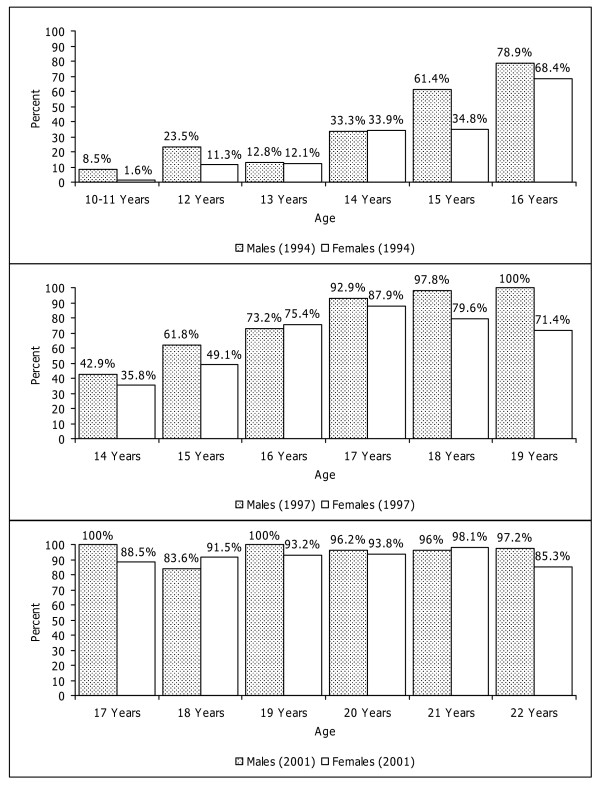
**Frequencies of alcohol consumption (≥ 1 glass of alcohol) at three times**.

## Pre-publication history

The pre-publication history for this paper can be accessed here:

http://www.biomedcentral.com/1471-244X/10/15/prepub
